# Neoadjuvant immunochemotherapy versus neoadjuvant immunoradiotherapy in locally advanced oral squamous cell carcinoma

**DOI:** 10.3389/fimmu.2025.1563737

**Published:** 2025-05-08

**Authors:** Gaofeng Ding, Wen Wang, Qingke Duan, Yufei Lu

**Affiliations:** Department of Radiotherapy, The Affiliated Cancer Hospital of Zhengzhou University & Henan Cancer Hospital, Zhengzhou, China

**Keywords:** neoadjuvant immunochemotherapy, neoadjuvant immunoradiotherapy, oral squamous cell carcinoma, pathologic complete response, quality of life

## Abstract

**Objective:**

To juxtapose the efficacy and safety profiles of neoadjuvant immunochemotherapy (NAIC) and neoadjuvant immunoradiotherapy (NAIR) in the management of locally advanced oral squamous cell carcinoma (SCC).

**Methods:**

A retrospective analysis of prospectively gathered data was conducted. The study evaluated the impact of NAIC versus NAIR on various parameters, including pathologic complete response (pCR), major pathologic response (mPR), clinical to pathological downstaging, surgical site infection, quality of life, pathologic adverse features, and prognostic outcomes.

**Results:**

The study encompassed a total of 120 patients, with 73 undergoing NAIC. The pCR and mPR rates in the NAIR group were 25.5% and 63.8%, respectively, closely mirroring the 31.5% and 69.9% observed in the NAIC cohort. A propensity for clinical to pathological downstaging and a reduced incidence of pathologic adverse features was noted in the NAIC population. However, both groups exhibited similar distributions in surgical site infection rates, quality of life metrics, grade 3/4 adverse events, and overall survival. In the Cox proportional hazards model, patients receiving NAIC demonstrated a hazard ratio of 0.87 (95% confidence interval: 0.65-0.98) for 3-year locoregional control, relative to the NAIR group.

**Conclusion:**

In the context of locally advanced oral SCC, both NAIC and NAIR exhibited robust efficacy and safety profiles. Nevertheless, NAIC provided superior locoregional control compared to NAIR, thereby emerging as the more favorable initial therapeutic option over NAIR.

## Introduction

Oral squamous cell carcinoma (SCC) ranks as the most prevalent malignant tumor among all head and neck cancers, with a majority presenting at an advanced stage upon initial diagnosis primarily due to lymph node metastasis (1). Current standard treatment consists of surgical intervention followed by adjuvant radiotherapy or chemoradiation; however, nearly half of these patients experience locoregional failure or distant metastasis (2). The lack of substantial improvement in prognosis underscores the pressing need for innovative treatment strategies for oral SCC.

In light of the encouraging survival advantages delineated by a seminal trial (3), immunotherapy has been sanctioned as the primary treatment modality for recurrent/metastatic SCC of the head and neck. A marked pivot towards exploring immunotherapy within the neoadjuvant context for untreated head and neck SCC has garnered considerable interest. A succession of clinical trials has demonstrated remarkable therapeutic efficacy and a paucity of adverse effects associated with neoadjuvant immunochemotherapy (NAIC) in head and neck SCC (4–6). In the Illuminate Trial (4), a cohort of twenty patients was enrolled. NAIC was found to be eminently tolerable, with a negligible incidence of grades 3-4 adverse events in but three patients. The rate of major pathological response (mPR) was 60%, encompassing a 30% pathological complete response (pCR). Throughout their median 23-month follow-up, disease-free survival was observed at 90%, with an overall survival (OS) rate of 95%. An additional phase II trial, involving 48 patients, yielded an objective response rate of 89.6%. Among the 27 patients who underwent surgical intervention, 17 (63.0%) achieved an mPR or pCR, with a pCR rate of 55.6%. Grade 3 or 4 treatment-related adverse events were reported in only two patients (5). A retrospective analysis of 21 patients (6) revealed an mPR of 66.7%, including 11 patients who attained a pCR. The overall response rate was 90.5%, and the rate of complete response was 28.6%. There were no grade 4 adverse events or instances of delayed surgery. Recently, a phase 1b trial concentrated on the efficacy of immunoradiotherapy (NAIR) in head and neck SCC (7), reporting mPR and pCR rates of 86% and 67% respectively. Clinical to pathological downstaging was observed in 90% of patients treated, with no delays in surgery. In another retrospective study (8), an analysis of 30 patients revealed no serious adverse events, with mPR, pCR, and clinical to pathological downstaging rates of 60.0%, 33.3%, and 83.3% respectively. Over a median follow-up period of 13.5 months, the disease-free survival and OS at 24 months were 70.4% and 76.4% respectively. Radiation oncologists are also keen to explore the synergistic potential of radiotherapy and immunotherapy in head and neck SCC (9). Current evidence suggests that both NAIC and NAIR demonstrate pronounced efficacy in head and neck SCC; however, the comparative effectiveness and safety profiles of these two modalities remain to be elucidated.

Thus, our objective is to compare the efficacy and safety profiles of NAIC and NAIR in the context of locally advanced oral SCC.

## Patients and methods

### Ethical approval

This study was approved by Our Hospital Institutional Research Committee, and written informed consent for medical research was obtained from all patients before starting the treatment. All methods were performed in accordance with the Declaration of Helsinki.

### Study design

To fulfill our objective, a retrospective analysis was conducted on prospectively collected data. Between January 2020 and December 2021 a total of 140 consecutive patients diagnosed with resectable cT1/2N+ or cT3/4N_any_ oral SCC were enrolled at a tertiary cancer center, but 20 cases refused to take part in this research. Finally, 47 patients received NAIR, while the remaining underwent NAIC. All participants were requested to complete the European Organization for Research and Treatment of Cancer Quality of Life Questionnaire (EORTC QLQ-C30) using the validated Chinese translation prior to neoadjuvant therapy, prior to surgery, six months postoperatively, and one year post-surgery. Patient demographics, pathology, treatment details, and follow-up information were meticulously analyzed.

### Study variables

All patients underwent contrast-enhanced MRI, CT, and PET/CT scans to assess the primary sites and the status of the neck. Tumor and neck stages were assessed according to the 8th edition of the AJCC system. All pathological specimens were reviewed by at least two experienced head and neck pathologists. The degree of pathological differentiation was classified into three categories: well-differentiated, moderately differentiated, and poorly differentiated. Lymphovascular invasion (LVI) was considered positive when tumor cells were detected within the lymphatic channels. Perineural invasion (PNI) was deemed present if tumor cells infiltrated nerve structures (10). mPR was defined as ≤ 10% residual viable tumor identified through pathological examination of the resected tissue, while pCR was characterized by the absence of residual malignant lesions (11). The combined positive score (CPS) served to evaluate the proportion of PD-L1-positive tumor cells and infiltrating immune cells relative to the total number of viable tumor cells.

The primary outcomes of interest included mPR and pCR. Secondary outcomes encompassed neoadjuvant therapy-related adverse events, clinical to pathological downstaging, quality of life (QoL), surgical site infection, adverse pathologic feature, 3-year locoregional control (LRC) and overall survival (OS). Locoregional control time was calculated from the date of surgery until the date of first locoregional recurrence or the last follow-up, while OS time was measured from the date of surgery to the date of death or the last follow-up. Radiologic responses were assessed in accordance with the Response Evaluation Criteria in Solid Tumors version 1.1, while adverse events were graded based on the NCI-CTCAE (version 4.0).

### NAIC, NAIR, and surgery

In the NAIC group, the treatment regimen included docetaxel at a dose of 75 mg/m², cisplatin at 75 mg/m², and pembrolizumab or alternative PD-L1 inhibitors at 200 mg of each three-week cycle by intravenous injection for two to three cycles. Conversely, the NAIR group received intravenous administration of Pembrolizumab or Penpulimab or Tislelizumab at 200 mg every two weeks. A prescribed dose of 40 Gy was delivered, targeting primary tumors and all radiographically visible metastatic lymph nodes. The target lesions were delineated and confirmed by two radiation oncologists as the gross tumor volume, which was then uniformly expanded by an additional 2–3 mm to establish the planning target volume. Radiation therapy was prescribed to ensure 95% coverage of the planning target volume, administered at 1.8–2.0 Gy per fraction, with five fractions per week.

Surgery was scheduled within one to four weeks following the completion of the neoadjuvant regimen. Surgical plans and resection margins were predefined based on baseline evaluations conducted prior to neoadjuvant therapy and remained unchanged irrespective of therapeutic response. Subsequent adjuvant therapy was initiated within six weeks post-surgery, focusing on the tumor bed with a margin of 1-2 cm. Adjuvant chemotherapy was administered based on clinical judgment and pathological characteristics, typically encompassing cisplatin over a duration of 4-6 cycles at a dose of 75 mg/m².

### EORTC QLQ-C30

The QLQ-C30 questionnaire has been transformed into five functional scales—physical, role, cognitive, emotional, and social—alongside three symptom scales that encompass fatigue, pain, and nausea/vomiting. Additionally, it includes a global health and QoL scale as well as six individual symptom measures. Patients were instructed to evaluate the presence of symptoms or functional limitations on a Likert scale ranging from one to four. A high score for the functioning scale and for the global QoL scale represents a better level of functioning, whereas higher levels in the symptom scales or the single-item scales denotes a high level of symptoms or problems.

### Statistical analysis

Primary outcomes were compared between the two cohorts utilizing the Chi-square test or Fisher’s exact test. LRC and OS was evaluated via univariate and Cox regression models, with results presented as hazard ratios (HR) and 95% confidence intervals (CI). Categorical secondary outcomes were analyzed using the Chi-square test or Fisher’s exact test, while continuous secondary outcomes were compared employing the Mann-Whitney U test. All statistical analyses were conducted using R version 3.4.4, and a p-value of less than 0.05 was deemed statistically significant.

## Results

### Baseline data

A total of 120 patients were included in this study, with a mean age of 55 ± 12 years. The cohort comprised 75 males and 45 females. The ECOG performance status was recorded as 0 in 53 patients and 1 in 67 patients. Among the participants, 67 were identified as smokers and 57 as drinkers. The primary tumor sites included the tongue in 49 patients, the floor of the mouth in 30 patients, buccal mucosa in 23 patients, and gingiva in 18 patients. Clinical stages were distributed as stage III in 77 patients and stage IV in 43 patients. A total of 20 patients had a CPS of less than 1, while 36 patients had a CPS of 20 or greater. Pathological differentiation was classified as well in 32 patients, moderate in 63 patients, and poor in 25 patients. Resection status of R0, R1, and R2 were accomplished in 115 (95.8%), 4 (3.3%), and 1 (0.8%) patients, respectively.

Seventy-three patients underwent NAIC, exhibiting a similar distribution across all variables compared to those receiving NAIR (all p > 0.05, **Table 1**). Of these 73 patients, 50 (72.6%) were administered two cycles of NAIC, while the remainder (27.4%) underwent three cycles of NAIC. Pembrolizumab, Penpulimab, and Tislelizumab were prescribed for 30 (41.1%), 20 (27.4%), and 23 (31.5%) patients, respectively.

**Table 1:** Demography and pathologic data between neoadjuvant immunochemotherapy (NAIC) and neoadjuvant immunoradiotherapy (NAIR) groups.

Variable	Total (n=120)	NAIC (n=73)	NAIR (n=47)	p*
Age				
≤55	60	33	27	
>55	60	40	20	0.190
Sex				
Male	75	45	30	
Female	45	28	17	0.809
ECOG PS^&^				
0	53	35	18	
1	67	38	29	0.299
Smoker				
No	53	31	22	
Yes	67	42	25	0.640
Drinker				
No	63	35	28	
Yes	57	38	19	0.213
Primary site				
Tongue	49	30	19	
Mouth floor	30	19	11	
Buccal	23	14	9	
Gingiva	18	10	8	0.961
Clinical stage				
III	77	45	32	
IV	43	28	15	0.473
CPS^#^				
<1	20	13	7	
1-20	64	36	28	
≥20	36	24	12	0.542
Differentiation				
Well	32	20	12	
Moderate	63	37	26	
Poor	25	16	9	0.878
Resection status				
R0	115	70	45	
R1	4	2	2	
R2	1	1	0	1.000

* refer to the comparison between NAIC and NAIR groups using the Chi-square test.

& ECOG, eastern cooperative oncology group performance status.

# CPS, Combined positive score.

### Primary outcome

In the NAIR group, mPR was observed in 63.8% of the total population, with 12 cases (25.5%) achieving a pCR. In the NAIC cohort, 51 patients demonstrated mPR, and pCR was noted in 23 cases (31.5%), although this difference was not statistically significant (p = 0.482). Patients in the NAIC group were more likely to achieve pCR.

**Figure 1:**
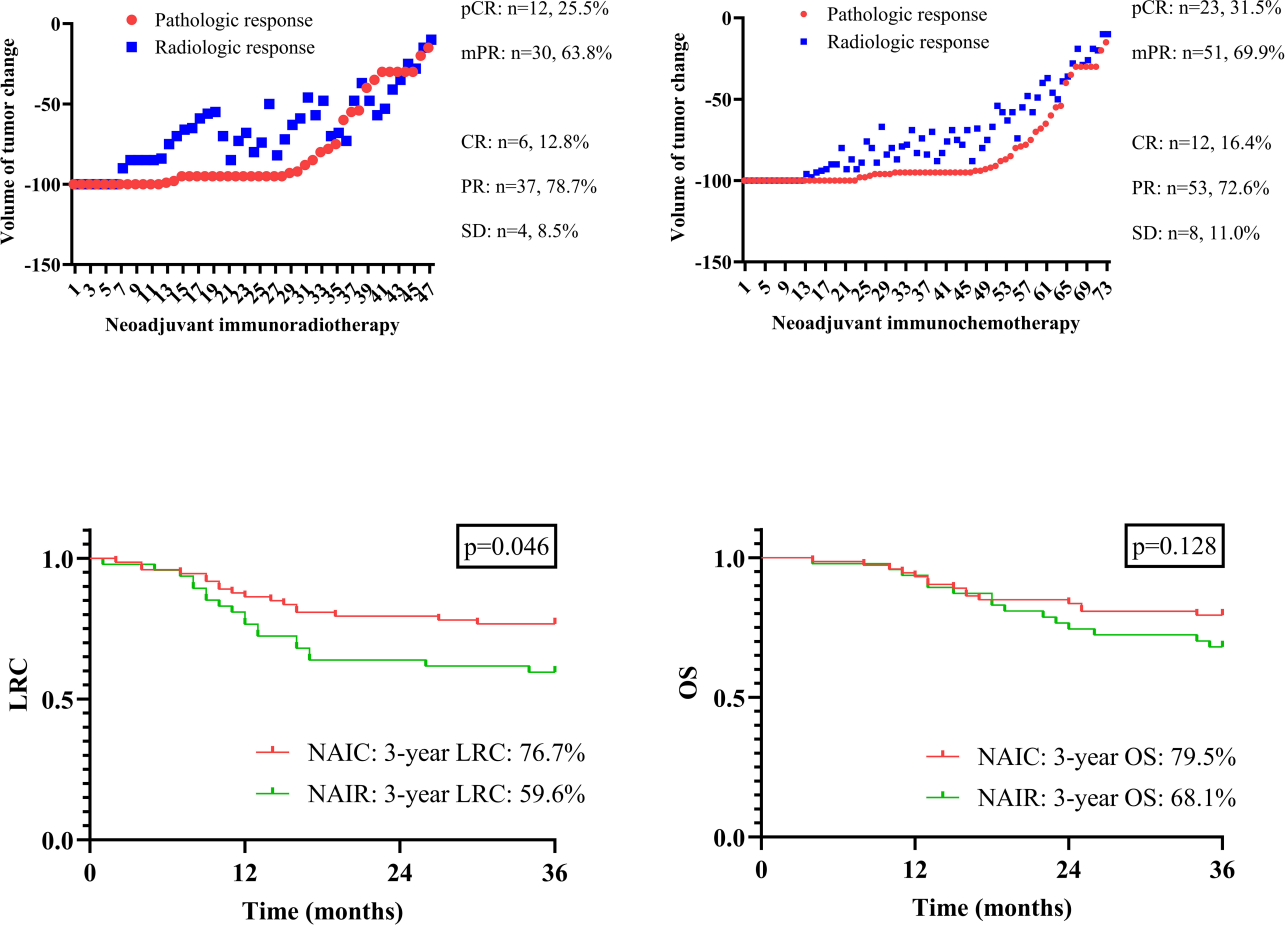
Radiologic and pathologic assessment in patients managed with neoadjuvant immunoradiotherapy and neoadjuvant immunochemotherapy; comparison of locoregional control (LRC) and overall survival (OS) in patients managed with neoadjuvant immunoradiotherapy and neoadjuvant immunochemotherapy.

The association between radiologic and pathologic assessments is illustrated in **Figure 1**. A pCR was consistently accompanied by a complete radiologic response; however, for other radiologic responses, the pathologic status could not be accurately predicted.

### Secondary outcome

**Figure 2:**
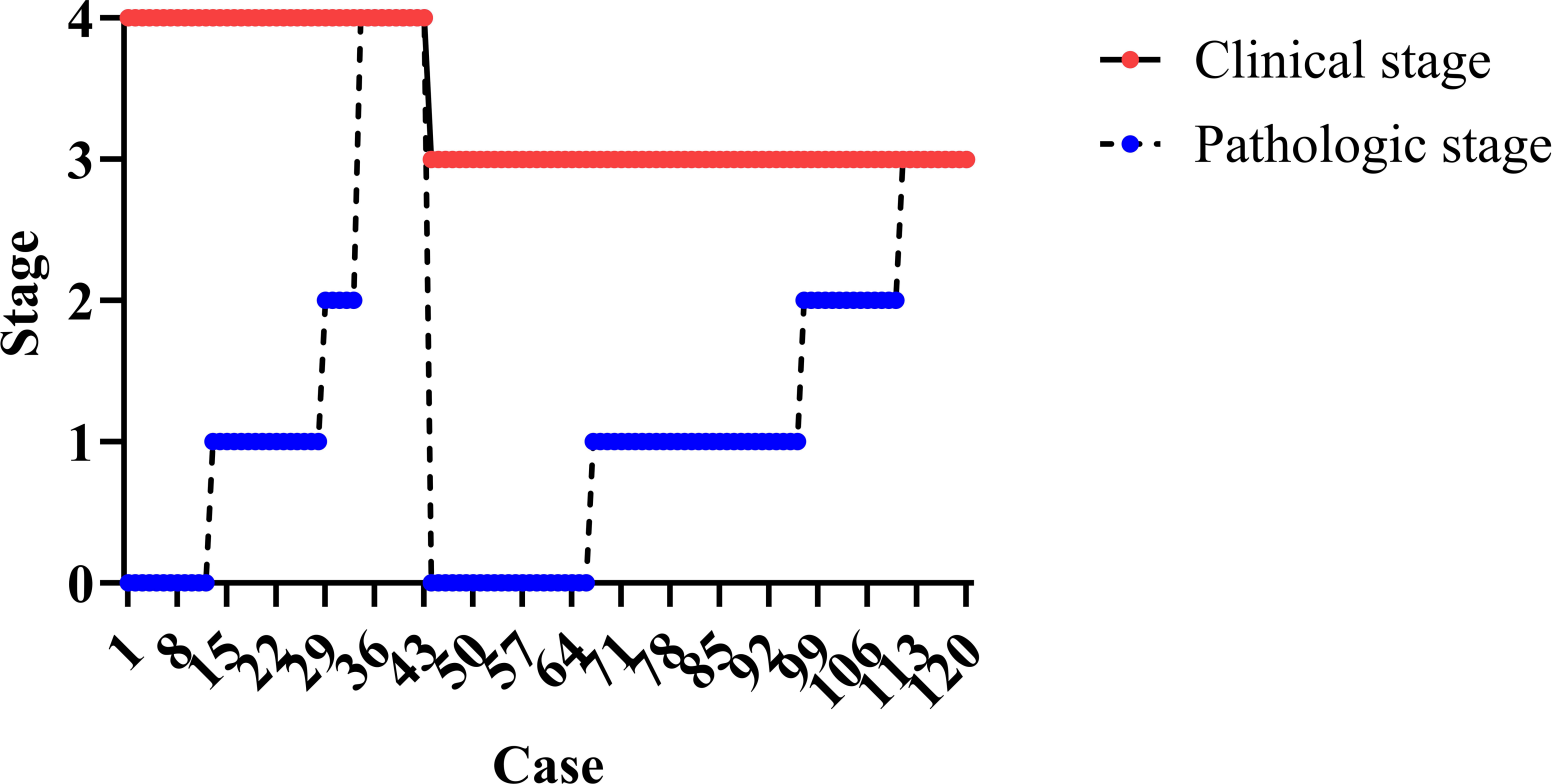
Detailed information of clinical to pathologic downstaging.

**Figure 3:**
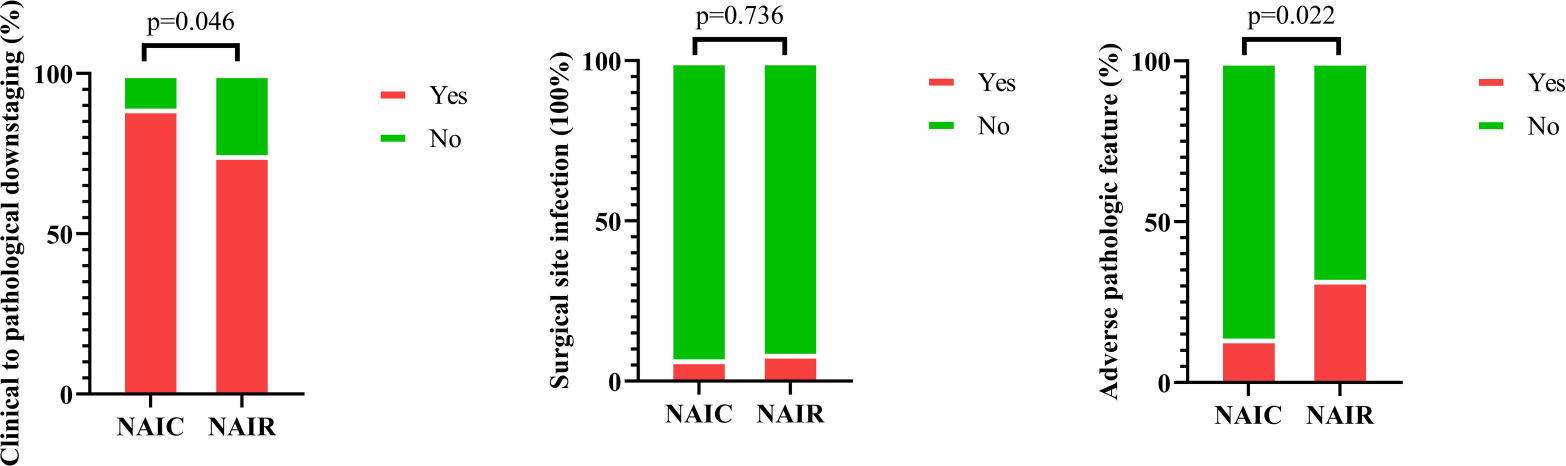
Comparison of incidences of clinical to pathologic downstaging, surgical site infection, and pathologic adverse features in patients managed with neoadjuvant immunoradiotherapy and neoadjuvant immunochemotherapy.

Clinical to pathologic downstaging was obtained in 100 patients (**Figure 2**), which was achieved in 65 patients (89.0%) in the NAIC group, significantly higher than the 74.5% observed in the NAIR population (p = 0.046). Adverse pathological features, including LVI, PNI, or extranodal extension, were noted in 13.7% of the NAIC group, which was significantly lower than the 31.9% in the NAIR cohort, the difference was mainly attributed by LVI distribution (**Table 2**, p = 0.022). The incidence of surgical site infections was similar between the two groups (6.8% vs. 8.5%, p = 0.736) (**Figure 3**).

**Table 2:** Adverse pathologic features in patients treated by neoadjuvant immunochemotherapy (NAIC) or neoadjuvant immunoradiotherapy (NAIR).

Adverse pathologic feature	NAIC (n=73)	NAIR (n=47)	p
Lymphovascular invasion	5 (6.8%)	9 (19.1%)	0.040
Perineural invasion	4 (5.5%)	7 (14.9%)	0.107
Extranodal extension	3 (4.1%)	6 (12.8%)	0.152
Overall	10 (13.7%)	15 (31.9%)	0.022

**Figure 4:**
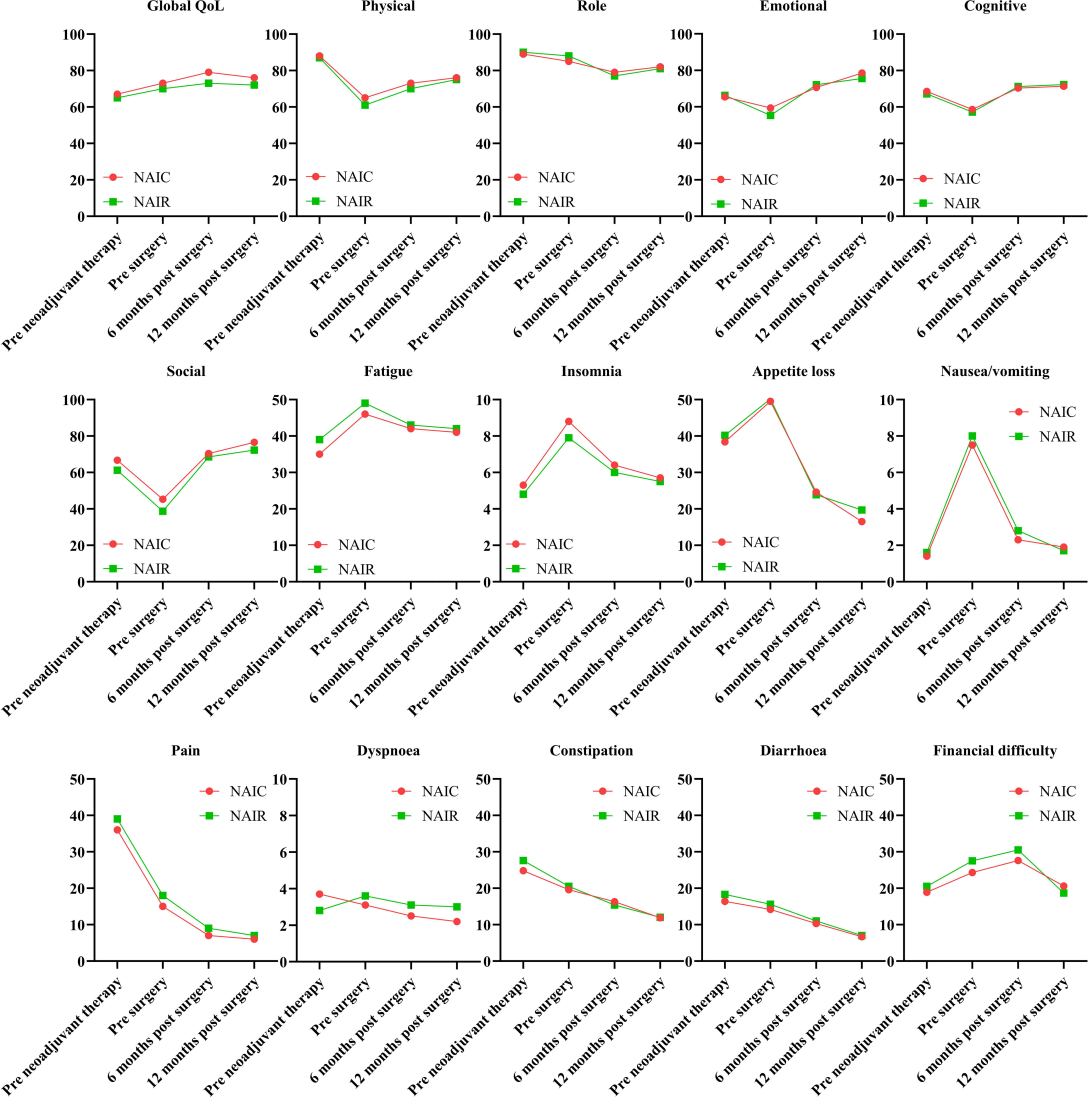
Quality of life in patients managed with neoadjuvant immunoradiotherapy and neoadjuvant immunochemotherapy.

The completion rate of the questionnaire was 100% in both groups prior to neoadjuvant therapy and before surgery. In the NAIC group, 70 patients (95.9%) and 60 patients (82.2%) completed the questionnaire at six months and twelve months postoperatively, respectively. In the NAIR population, 41 patients (87.2%) and 40 patients (85.1%) completed the questionnaire at six months and twelve months postoperatively. Global QoL showed continuous improvement from the onset of therapy, maintaining a stable status at six months post-surgery. All five functional scales exhibited significant declines following the completion of neoadjuvant therapy but gradually returned to baseline levels or improved within six months post-surgery. Symptoms displayed dynamic alterations at various time points, with complaints of pain, constipation, and diarrhea consistently decreasing. No significant differences were observed across all domains between the two cohorts at the same time points (all p > 0.05, **Figure 4**).

Neoadjuvant therapy-related adverse events were prevalent, though most were graded as 1 or 2. The most common grade 3/4 event in both groups was mucositis, followed by rash and anemia, with both cohorts exhibiting similar incidences of all grade 3/4 events (all p > 0.05, **Table 3**).

**Table 3:** Neoadjuvant therapy related adverse events in neoadjuvant immunochemotherapy and neoadjuvant immunoradiotherapy groups.

Event	NAIC (n=73)		NAIR (n=47)		p*
	Grade 1/2	Grade 3/4	Grade 1/2	Grade 3/4	
Mucositis	47 (64.4%)	4 (5.5%)	30 (63.8%)	3 (6.4%)	1.000
Vomiting	43 (58.9%)		30 (63.8%)		
Xerostomia	34 (46.6%)		25 (53.2%)		
Fatigue	30 (41.1%)		21 (44.7%)		
Rash	25 (34.2%)	3 (4.1%)	20 (42.6%)	2 (4.3%)	1.000
Pain	24 (32.9%)		19 (40.4%)		
Hypotension	19 (26.0%)		16 (34.0%)		
Anemia	13 (17.8%)	2 (2.7%)	11 (23.4%)	1 (2.1%)	1.000
Anorexia	11 (15.1%)		9 (19.1%)		
Hypothyroidism	11 (15.1%)		9 (19.1%)		
Leukopenia	11 (15.1%)	1 (1.4%)	8 (17.0%)	1 (2.1%)	1.000
Hypokalemia	9 (12.3%)		8 (17.0%)		
Transaminitis	7 (9.6%)	1 (1.4%)	7 (14.9%)	0	1.000
Fever	5 (6.8%)		6 (12.8%)		
Hyponatremia	4 (5.5%)		5 (10.6%)		
Pneumonia	2 (2.7%)		1 (2.1%)		

*refer to the comparison of grade 3/4 event incidence between the two groups using the Fisher test.

All patients were followed for at least three years, during which 36 locoregional recurrences and 30 deaths were documented. The three-year OS rates were 79.5% in the NAIC group and 68.1% in the NAIR group, although this difference was not statistically significant (p = 0.128, **Figure 1**). However, the NAIC cohort demonstrated a three-year LRC rate of 76.7%, which was significantly higher than the 59.6% observed in the NAIR group (p = 0.046, **Figure 1**).

To assess the independence of these findings, a Cox regression model was performed, incorporating neoadjuvant therapy and yp stage as factors due to their significance in the univariate analysis. Compared to the NAIR group, patients receiving NAIC had a HR of 0.87 (95% CI: 0.65-0.98). When comparing patients with a yp T0N0 stage, those with yp stages I/II did not show an increased risk of locoregional failure. However, patients with yp stages III/IV exhibited a significantly higher risk, with an HR of 4.47 (95% CI: 2.10-12.45) (**Table 4**).

**Table 4:** Cox model analysis the impact of neoadjuvant immunochemotherapy (NAIC) versus neoadjuvant immunoradiotherapy (NAIR) on locoregional control.

Variable	p	HR [95%CI]
Neoadjuvant therapy (NAIC vs NAIR)	0.032	0.87 [0.65-0.98]
yp stage		
ypT0N0		ref
yp stage I/II	0.218	2.86 [0.56-7.59]
yp stage III/IV	0.011	4.47 [2.10-12.45]

## Discussion

Our paramount discovery entailed that in the context of locally advanced oral SCC, NAIC and NAIR exhibited comparable efficacy and safety, manifesting satisfactory rates of pCR and mPR, along with a low incidence of grade 3/4 adverse events. Nonetheless, NAIC not only afforded a superior three-year LRC but also yielded a greater likelihood of clinical to pathological downstaging and a reduced prevalence of adverse pathological features compared to NAIR. QoL was significantly affected by neoadjuvant therapy, yet nearly all scales experienced a recovery to baseline levels or achieved even better status. This investigation stands as the inaugural study to compare NAIC and NAIR in the treatment of locally advanced oral SCC, thereby elucidating a preference for NAIC as the more favorable treatment option over NAIR.

In light of the promising survival benefits associated with immunotherapy in the recurrent/metastatic setting of head and neck SCC (3), the potential of immunotherapy as a neoadjuvant treatment has garnered considerable interest, with a multitude of clinical investigations having been reported. A recent systematic review (12) collated data from 1092 patients across 24 studies, revealing an average objective response rate of 37%. Notably, immunochemotherapy demonstrated a superior objective response rate compared to immunotherapy alone in patients with untreated head and neck SCC. Therefore, the combination of immunotherapy with other therapeutic modalities tended to elicit a more efficacious response than immunotherapy administered in isolation. In a preceding phase 1b clinical trial (7), a cohort of twenty-one patients underwent treatment with NAIR at a cumulative dose of either 40 Gy administered in five fractions or 24 Gy in three fractions. All patients tolerated the treatment well, with no resultant delays in surgery. Within this collective study population, the rates of mPR and pCR were 86% and 67%, respectively. Clinical to pathological downstaging was observed in 90% of the treated patients. This outcome was particularly striking, as the majority achieved a pCR, which is indicative of a longer survival duration. However, a notably lower incidence of pCR was observed in the present study, with a potential explanation being that we exclusively enrolled patients with oral SCC, whereas the previous study comprised predominantly of patients with HPV-positive oropharyngeal SCC, a subset known to respond favorably to radiotherapy. Another retrospective investigation (9) delineated the outcomes of 30 oral SCC patients who received NAIR, with all cases demonstrating good tolerance to the neoadjuvant treatment, devoid of serious adverse events. The rates of complete response, partial response, and stable disease were 10.0%, 46.7%, and 43.3%, respectively. The rates of mPR, pCR, and clinical to pathological downstaging were 60.0%, 33.3%, and 83.3%, respectively. Over a median follow-up period of 13.5 months, 26 patients (86.7%) who had undergone surgical resection remained alive. The disease-free survival and OS at 24 months were 70.4% and 76.4%, respectively. These findings, in conjunction with our own depiction, collectively underscore the high efficacy and safety profile of NAIR in the treatment of oral SCC.

Contemporary literature increasingly favors the concomitant use of immunotherapy and chemotherapy as a neoadjuvant treatment regimen. Huang et al. (4) enrolled 20 patients with locally advanced oral SCC, wherein NAIC was well-tolerated, with only three patients experiencing grades 3-4 adverse events. The completion rates for NAIC and subsequent R0 resection were uniformly 100%. The mPR rate stood at 60%, encompassing a 30% pCR. Over a median follow-up period of 23 months, disease-free survival and OS rates were 90% and 95%, respectively. Yao et al. (6) presented an analysis of 21 patients with head and neck SCC who underwent radical surgery and comprehensive cervical lymph node dissection following NAIC. The mPR rate was 66.7%, with 11 patients achieving a pCR. The overall response rate was 90.5%, and the complete response rate was 28.6%. The predominant adverse event was anemia, occurring in 61.9% of patients. No grade 4 adverse events or surgical delays were reported. Laryngeal preservation rates reached 90.9%, and all patients had negative surgical margins confirmed pathologically. In a separate cohort of 79 patients reported by Yan et al. (13), the R0 resection rate was an impressive 98.7%. Pathological assessment revealed that 53.1% of patients achieved either pCR or mPR. Following a median follow-up of 17.0 months, the 1-year disease-free survival and OS rates were 87.2% and 97.4%, respectively. Comparable findings were also corroborated by Chen et al. (14), Yu et al. (15), and our own analysis. Significantly, our study may be the first to address the question of whether there exists a discernible difference in efficacy and safety between NAIC and NAIR. On the one hand, both treatment arms demonstrated high pCR and mPR rates, with no substantial disparity in surgical site infection rates or overall survival. On the other hand, NAIR was associated with a less favorable 3-year LRC, a finding that may be attributed to a reduced likelihood of clinical to pathological downstaging and a higher prevalence of adverse pathologic features in patients treated with NAIR.

QoL constitutes a pivotal consideration in the management of cancer (16), yet regrettably, it is seldom analyzed in the aftermath of neoadjuvant therapy. To the best of our knowledge, only a single pertinent study has been documented. In this study (11), 30 patients with oral SCC treated with NAIR were assessed. Regarding the functional scales, emotional, physical, social, role, and cognitive functioning demonstrated improvement at 1.5 and 2 years post-radiotherapy completion, with all functional scores equating to or surpassing baseline levels at the 2-year mark. All EORTC QLQ-C30 functioning and symptom scales, excluding nausea and vomiting, exhibited significant resolution at 2 years following the conclusion of radiotherapy. These findings align with those observed in our NAIR cohort, albeit we have conducted a comparative analysis between NAIC and NAIR. On the one hand, it was observed that the impact of both interventions on each QoL domain was analogous at corresponding time points. On the other hand, it was intriguing to note that while global QoL consistently recovered, other functional and symptom scales—except for pain, constipation, and diarrhea—experienced a minor deterioration following the completion of neoadjuvant therapy. This observation is reflective of the efficacy in cancer control exhibited by both NAIR and NAIC.

Limitation in current study must be acknowledged, first, there was lack of randomization, it increased our selective bias; second, our sample size was relatively small, it might decrease our statistic power; third, this was a single-center design limited by relatively short follow-up, further clarification on long-term toxicities, biomarker-driven stratification, and external validation was needed.

In conclusion, within the context of locally advanced oral SCC, both NAIC and NAIR demonstrated substantial efficacy and safety, characterized by comparable rates of pCR and mPR, as well as analogous QoL and OS. However, NAIC conferred a superior LRC compared to NAIR, thereby positioning NAIC as the preferable initial therapeutic choice over NAIR.

## Data Availability

The original contributions presented in the study are included in the article/Supplementary Material. Further inquiries can be directed to the corresponding author/s.
